# Relationship of family caregiver burden with quality of care and psychopathology in a sample of Arab subjects with schizophrenia 

**DOI:** 10.1186/1471-244X-10-71

**Published:** 2010-09-10

**Authors:** Muhammad A Zahid, Jude U Ohaeri

**Affiliations:** 1 Department of Psychiatry, Faculty of Medicine, Kuwait University; P.O. Box 24923, Safat, 13110, Kuwait; 2 Department of Psychiatry, Psychological Medicine Hospital, Kuwait

## Abstract

**Background:**

Although the burden experienced by families of people with schizophrenia has long been recognized as one of the most important consequences of the disorder, there are no reports from the Arab world. Following the example of the five - nation European (EPSILON) study, we explored the following research question: How does the relationship between domains of caregiving (as in the Involvement Evaluation Questionnaire - IEQ-EU) and caregiver psychic distress on the one hand, and caregiver's/patient's socio-demographics, clinical features and indices of quality of care, on the other hand, compare with the pattern in the literature?

**Method:**

Consecutive family caregivers of outpatients with schizophrenia were interviewed with the IEQ-EU. Patients were interviewed with measures of needs for care, service satisfaction, quality of life (QOL) and psychopathology.

**Results:**

There were 121 caregivers (66.1% men, aged 39.8). The IEQ domain scores (total: 46.9; tension: 13.4; supervision: 7.9; worrying: 12.9; and urging: 16.4) were in the middle of the range for the EU data. In regression analyses, higher burden subscale scores were variously associated with caregiver lower level of education, patient's female gender and younger age, as well as patient's lower subjective QOL and needs for hospital care, and not involving the patient in outdoor activities. Disruptive behavior was the greatest determinant of global rating of burden.

**Conclusion:**

Our results indicate that, despite differences in service set-up and culture, the IEQ-EU can be used in Kuwait as it has been used in the western world, to describe the pattern of scores on the dimensions of caregiving. Differences with the international data reflect peculiarities of culture and type of service. Despite generous national social welfare provisions, experience of burden was the norm and was significantly associated with patient's disruptive behavior. The results underscore the need for provision of community - based programs and continued intervention with the families in order to improve the quality of care.

## Background

The term family or informal caregiver burden refers to the physical, psychological and social impact that caring for relatives with chronic disorders has on families [1,2]. Some authors prefer the term "family caregiving consequences" because many family members express positive emotions about the experience [2]. In this paper, we use the terms "burden" and "caregiving consequences" interchangeably. Objective burden is defined as the observable costs to the family that result from the disease (such as disruption to everyday life). Subjective burden includes the individual's perception of the situation as burdensome [3]. Although the burden experienced by families of people with schizophrenia has long been recognized as one of the most important consequences of the disorder[2,4], there is a divergence of opinion about associated factors and effectiveness of intervention measures[1,5,6].

The only reports on family burden in psychiatry from the Middle East are from Iran [7,8]. The rationale for this report is that we do not know the pattern of family caregiver consequences in the Arab world and how this compares with the situation in other parts of the world. Since family burden may be influenced by differences in mental health service provisions, social network and other cultural factors [9-12], it is important that the generalizability of findings be tested in various contexts [13]. Hence, while family burden gained attention in the Western world largely because of the emphasis on de - institutionalization and the consequent establishment of community-based programs [1], it should be interesting to compare with the situation in an Arab country like Kuwait which has never had a history of formal institutionalization, and there are no community-based programs for the mentally ill. In other words, while the extent of family burden has become an essential indicator for mental health service provision in the western world, family burden can be viewed as the non-mediated effect on families of living with and caring for a relative affected by schizophrenia in Kuwait and countries with similar mental health service history. Furthermore, the social network is vastly different from the western world [14]. In this regard, we have sought to link family burden with schizophrenia patients' socio-demographic characteristics, quality of care and psychopathology, because these are the factors that have been mostly reported to be associated with severity of burden [1,2]. Whereas there are numerous reports on the association with socio-demographic and clinical characteristics [6], there is a paucity of studies on the relationship with indices of quality of care (such as patients' met/unmet needs for care, service satisfaction and quality of life) [13,15-18].

Of the variables investigated, there is more agreement that severer levels of burden are associated with lower levels of caregiver education [19-21], patient's problematic or disruptive behavior, and positive and negative symptoms of schizophrenia [13,22,23]. Rarely, psychopathology and duration of illness were found not to be significantly associated with burden [24]. While most reports indicated that higher levels of burden were associated with caring for patients who were male, younger, with longer duration of illness and with whom they had more contact [11,17,21,25,26], few reported that burden was either associated with caring for females[23] or had no significant association with sociodemographic characteristics[27], and duration of illness [28].

Following the example of the five - nation European Psychiatric Services: Inputs Linked to Outcome Domains and Needs (EPSILON) study [12], we have used the responses of a sample of Kuwaiti schizophrenia family caregivers to the Involvement Evaluation Questionnaire (IEQ-EU) [29], to examine the above issues by exploring the following research question: How does the relationship between IEQ-EU domains of caregiving and caregiver psychic distress on the one hand, and caregiver's/patient's socio-demographic characteristics, clinical features and indices of quality of care, on the other hand, compare with the pattern in the literature [12,28,30,31]?

In addressing the issues, our conceptual framework was based on Schene's [32] theoretical model, namely: the chronic illness of a family member is considered as an objective stressor that because of the caregiving role results in strain for the family caregiver. The impact of caregiving on the caregiver depends on the characteristics of the patient, the caregiver, their relationship and the environment (e.g., quality of hospital care).

## Method

### The setting

Kuwait is a city - state located in the Arabian Gulf (population, 3.4 million). For Kuwaiti nationals, there is an effective national social welfare system. The country has a conservative Muslim culture, with traditional gender roles and sexual segregation, and the extended family system and family social support are the norms.

The study was carried out at the Psychological Medicine Hospital, the only facility of its kind in Kuwait (691 in - patient beds). There are no community - based mental health care services, such as home visits, crisis intervention, sheltered accommodation and sheltered work. All services provided to Kuwaiti nationals are free - of - charge. Health care delivery is sectorized (i.e., in catchment areas). There are five general adult psychiatric catchment area units. Patients involved in this study belonged to the catchment area of one of us (MAZ).

### Participants

The participants were principal family caregivers of consecutive attendees at the unit, who fulfilled the study's inclusion criteria. We sought to have patients with comparable characteristics to those of the EPSILON study. Hence, the patients selected were attending follow - up clinic appointment, in stable clinical condition, had been ill for at least one year, aged less than 65 years, literate in Arabic, and could independently provide informed consent to participate. In addition, they were accompanied by family caregivers who lived with them. All patients had a stable (at least one year) case note diagnosis of schizophrenia, which was verified by the administration of the ICD-10 Symptom Checklist as in the Schedule for Clinical Assessment in Neuropsychiatry [33]. There were 130 patients (68.5% men, aged 14-61 yrs, mean 36.8, SD 10), and 95.5% were living together with either their spouses or families of origin [14]. Their general level of psychosocial functioning was average (GAF score = 50.2), while the mean BPRS (18 - item) score of 44.4 indicated that they were clinically "moderately ill"[34]. This report concerns the responses of 121 family caregivers (66.1% men, mean age 39.8, SD 12.6), because nine family caregivers did not attend appointment to complete the interviews. The disproportionate representation of men is due to the strict cultural rule that limits the role of women outside the home, including bringing patients to hospital. However, all the subjects interviewed for this report were the primary caregivers at home.

Of the 121 participants, 101 (83.5%) were either parents, children or siblings of the patients (Table 1).

**Table 1:** Socio-demograhic characteristics of caregivers and IEQ overall burden: N = 121

**Variables**	**N**	**%**
Age range (yrs): 17 - 3 0	35	28.9
31-40	31	25.6
41-75	55	45.5
Mean(SD): 39.75(12.6); Median: 39; Mode: 38		
Sex: Men	80	66.1
Women	41	33.9
Marital status: Single	23	19.0
Married	85	70.2
Divorced	13	10.7
Living arrangements: Patient lives with spouse/own children	74	61.2
Patient lives with parents/siblings	41	33.9
Patient lives with other relatives: uncle/aunt/cousin	6	5.0
Relationship of caregiver with patient: Parent	20	16.5
Patient's own child	22	18.2
Sibling	59	48.8
Spouse	9	7.4
Other relatives	11	9.1
Number of family members living in same household: < 6	37	30.6
6 - 9	60	49.6
>/= 10	24	19.8
Family monthly income: < KD 250 (USD 850.00)	15	12.4
KD 250 - 499	33	27.3
KD 500 - 999	55	45.5
>/= 1000.00 (USD 3400.00)	18	14.9
Average weekly telephone/personal contact: 1 - 4 hrs	23	19.0
5 - 8 hrs	13	10.7
9 - 16 hrs	16	13.2
17 - 32 hrs	15	12.4
> 32 hrs	54	44.6
IEQ43: Global subjective burden: Is relative's problem a burden		
past 4 wks?: No burden at all	8	6.6
A slight burden	24	19.8
A fairly heavy burden	34	28.1
A heavy burden	32	26.4
A very heavy burden	23	19.0

### Ethical approval

Ethical approval for the work was obtained from the Research and Ethical Committee of the Faculty of Medicine, Kuwait University. Patients and family caregivers gave verbal informed consent after the objectives of the study had been explained to them. As is well known in this culture for such non-invasive studies [35], all families approached freely consented to participate.

### Assessment instruments

The subjects were assessed with the instruments used for the EPSILON study, namely: the European (EU) versions of: (i) the Involvement Evaluation Questionnaire - for the relatives (IEQ-EU)[29]; (ii) the Verona Service Satisfaction Scale (VSS-EU) [36]; (iii) the Camberwell Assessment of Need (CAN-EU) [37]; and (iv) the Lancashire Quality of Life Profile (LQoLP-EU) [38]. The process of modifying all these instruments to suit the Kuwaiti situation and translating them into Arabic (from the English version) by the method of back - translation has been described elsewhere [14]. Psychopathology was assessed with the following: 14 items of the ICD-10 Symptom Checklist [33]; the 24-item version of the Brief Psychiatric Rating Scale (BPRS) [39]; and the Global Assessment of Functioning scale (GAF) [40].

We obtained all the assessment instruments of the EPSILON study from the authors [41]. Only the IEQ - EU will be described in detail here because it is the focus of this report. Details about the other questionnaires have been presented elsewhere [14,42].

### Caregiving consequences

The IEQ-EU [29] is an 81-item instrument that measures the consequences of psychiatric disorders for relatives of patients in the past four weeks. Of the 31 items on caregiving consequences, 27 are grouped into four subscales, namely, "tension" (9 items), "worrying" (6 items), "urging" (8 items), and "supervision" (6 items). In addition, a 27-item total score (summed score for IEQ items 16-35 and 37-43) can be computed. However, two items (IEQ29 on sleep disturbance, and IEQ43 on global subjective rating of burden) are each included in two scales, based on a previous factor analysis report [30]. IEQ29 is included in "tension" and "supervision", while IEQ43 is included in "tension" and "worrying". These items are rated either on a five-point (0-4) Likert scale, or a categorical (never/sometimes = 0; and regularly/often/always = 1) scale. The four items not included in the four subscales are IEQ36 (ability to pursue own activities), IEQ44 (getting used to patient's problems), IEQ45 (ability to cope with patient's problems) and IEQ46 (change in emotional relationship).

The 12 - item Goldberg's General Health Questionnaire [43] is included as a measure of caregiver severity of psychic distress, not as a screening instrument for mental disorders. The other sections of the IEQ include items on caregiver use of professional help, financial costs and consequences for the patient's children.

Although it is recommended that the IEQ should be self-administered, all assessments were interview - based, and carried out in Arabic by two experienced Arab specialist psychiatrists, because of the length of the questionnaire and the need to ensure that the participants understood the questions. The IEQ did not require modification for use in our setting because, in a review at the preliminary stage of the study, it was judged that the questionnaire is framed in culture - neutral language, and addresses caregiver experiences that are universal [29,31].

After a period of training, we performed inter-rater reliability tests for the two assessors using the responses of subjects who did not participate in the main study (the results will be presented elsewhere). Using the responses of the 121 participants in the main study, the internal consistency (Cronbach's alpha) of the 27 items of the IEQ were as follows: (i) tension subscale: 0.91; (ii) supervision: 0.81; (iii) worrying: 0.79; (iv) urging: 0.89; (v) total 27 items: 0.93.

We analyzed the data on the subscale scores and the 27 items in two ways. First, we used the summed scores for the susbscales based on the Likert scale (0-4) for univariate and multivariate statistical analyses, as recommended [29]. Also as recommended [30], we computed the scores based on the categorical scale and used this only for comparing the pattern of scores with some international data [12,25,44]. Second, based on the definition of subjective burden earlier highlighted [3], we chose IEQ43 to represent subjective burden, and included this item as a dependent variable in the subsequent analyses. In view of this, and following on the definition of objective burden earlier highlighted [3], we computed an additional total objective burden score consisting of 26 items (i.e., minus IEQ43) and included it as a dependent variable. The Cronbach's alpha for these 26 items was 0.93. We emphasize that all the subscale and total score comparisons with the international data were based on the original 27 - item scale.

### Service satisfaction

The VSSS-EU [36] is a 54 - item instrument that consists of seven domains, namely: global/overall satisfaction; professional skill and behaviour; information; access; efficacy; types of intervention; and relatives' support.

### Lancashire Quality of Life Profile (LQoLP-EU)

The LQoLP-EU [38], a structured interviewer - administered instrument, is a widely used instrument for the assessment of QOL in schizophrenia research [45]. It combines objective, factual, information related to several different life domains (i.e., objective QOL indicators) with subjective satisfaction with those domains (i.e., subjective QOL indicators). The objective components are evaluated on a scale of: Yes/No/Don't know. The questionnaire allows for the assessment of the following additional areas: (a) five positive items for positive affect and five negative items for negative affect from the Bradburn Scale [46]; and (b) the 10 - item Rosenberg Self-esteem scale [47].

### Patients' met/unmet needs

The CAN-EU [37], an interviewer - administered instrument, assesses patients' needs as perceived by them (as users) and the staff who have knowledge of them. It comprises 22 items of met and unmet needs. The scores of the 22 items are gathered into five groups of met and unmet needs, namely: basic (3 items), health (7 items), social (3 items), functioning (5 items), and service (4 items).

### Data collection procedure

At the preliminary stage of the study, the research team scrutinized the questionnaires for appropriateness of content in the Kuwaiti setting. Two native Arabs, who are fluent in English, jointly produced the Arabic translations of the instruments by the method of back - translation. Thereafter, one of us (MAZ), an experienced British - trained psychiatrist, trained two Arab specialist psychiatrists in the use of the questionnaires. Assessments generally took place at intervals over a period of one week, to suit the convenience of the families.

### Data analysis

Data were analyzed by the SPSS 15 (SPSS Inc., Chicago, Illinois). We used parametric statistics (t-tests, one-way ANOVA and Pearson's correlation) because the IEQ subscale scores were fairly normally distributed.

### Dependent and independent variables

In order to assess the factors associated with caregiver burden, the following were used as dependent variables: (i) the four IEQ subscales scores; (ii) the summed score of the 27 items; (iii) item IEQ43 on global rating of subjective burden; and (iv) total objective burden, computed from 26 items as highlighted above. All the others were treated as independent variables. In view of the many significant relationships in univariate analyses, we used step - wise regression analyses. Based on previous studies [30], the independent variables were entered in the following steps: (i) Step 1: background caregiver's and patient's socio-demographic characteristics; (ii) Step 2: Patient's CAN and VSSS scores; (iii) Step 3: Patient's LQLP subjective QOL, affect and self-esteem scores; (iv) Step 4: LQLP objective QOL indices; (v) Step 5: patient's psychopathology and GAF scores. For the multiple regression data, multi-collinearity was assessed by the values of "tolerance" (cut-off score </= 0.2) and variance inflation factor (VIF - cut-off score >4.0) [48]. Our IEQ mean subscale scores were compared with those of other studies by using effect size calculations. The level of statistical significance was set at P < 0.05.

## Results

### Pattern of subscale scores (Tables 1 and 2)

**Table 2:** IEQ subscale scores and frequency of items not in subscales: N = 121

IEQ subscales/items	Mean* (SD)	Possible range*	Actual range	Mean** (SD)	Range
Tension: 9 items***	13.4(7.6)	0-36	1-33	3.8(3.2)	0-9
Supervision: 6 items	7.9(4.9)	0-24	0-21	2.2(1.9)	0-6
Worrying: 6 items	12.9(5.0)	0-24	0-24	3.8(1.8)	0-6
Urging: 8 items	16.4(7.5)	0-32	0-32	4.7(2.6)	0-8
Overall score: Items: 16-35; 37-43.	46.9(19.2)	0-108	5-98	13.4(7.3)	0-27
Total objective burden: 16-35; 37-42	44.6(18.6)	0-104	4-95		
Caregiver GHQ-12 score	4.5(4.2)	0-12	0-12		
Frequency of items not included in subscales	Never (%)	Sometimes (%)	Regularly (%)	Often (%)	Always (%)
IEQ36: Able to pursue activities	6(5.0)	42(34.7)	37(30.6)	36(29.8)	-
IEQ45: Felt able to cope with pt	6(5.0)	31(25.6)	36(29.8)	26(21.5)	22(18)
	No	A little	Fairly	Very	A lot
IEQ44: Got used to pt's problem	5(4.1)	18(14.9)	34(28.1)	41(33.9)	23(19)
IEQ46: relationship with patient changed since illness	22(18.2)	37(30.6)	29(24.0)	20(16.5)	13(10.7)

* Using Likert scale response option: 0-4 (van Wijngaarden et al 2000)

** Using categorized scale: 0 - 1 (van Wijngaarden et al 2003).

*** IEQ29 is included in "tension" and "supervision"; IEQ43 is included in "tension" and "worrying"

The mean and median of the subscale scores indicated that the majority of caregivers had mostly average burden experience. In line with this, 89 (73.5%) rated the global burden of care as "fairly - very" heavy (Table 1), and 60.4% indicated that their caring role had affected their ability to pursue their activities "regularly - always". Nevertheless, 81.0% indicated that they had got used to the patients' problems ("fairly - a lot"). The mean GHQ-12 score (using the 0/1 scoring method) was 4.5 (and for the 1-4 scoring method: 15.1, SD 7.0).

In comparison with the pooled data for the EPSILON study [29], while our subjects tended to have lower subscale scores (except urging), this tendency reached significance for only the total score (Effect size = 0.21, 95% C.I.: 0.00 - 0.43). In individual comparison with the scores for the five nations, however, our scores were in the middle of the range for the Europeans. Our subjects shared similarity of scores on a number of subscales, namely, with Amsterdam (for total score and supervision), with Santander (for tension), with Copenhagen (for worrying) and with London and Verona (for urging). Our mean subscale scores for total, worrying and supervision were lower than those for Hong Kong, while being higher than Hong Kong for urging [28]. Interestingly, our GHQ-12 score was significantly much lower than that for Hong Kong (Effect size: 1.42, 95% C.I. = 1.16 - 1.67). Following the example of the EPSILON and Hong Kong data, we did not analyze the GHQ-12 data by cut-off scores, because it was not used as a screening instrument.

### Factors associated with caregiver burden

#### Relationship with family caregiver's characteristics

Caregiver GHQ-12 and objective burden indices were not significantly associated with their sex, age, marital status, living arrangements, family income, and duration of weekly personal contacts (P > 0.05). The tendency for caregivers with primary school level education to have higher objective burden scores than those with higher levels of educational attainment, reached significance for the following: (i) tension subscale (F = 6.9, df = 2/118, P = 0.001); (ii) worrying subscale (F = 7.4, P = 0.001); (iii) supervision subscale (F = 4.2, P < 0.018); and (iv) total objective burden score (F = 4.8, P < 0.01).

### Association with kinship relationship

Caregivers who were either children or spouses of patients had a tendency to have higher burden scores than other relationship groups. This trend reached significance for the following: (i) urging subscale: F = 6.1, df = 4/116, P < 0.001; (ii) total score: F = 3.1, P < 0.02; and (iii) worrying: F = 2.9, P < 0.03.

### Relationship of financial expenditure with caregiver burden and GHQ-12 scores

Financial expenditure was consistently associated with objective/subjective burden and GHQ-12 scores. Those who incurred the highest extra expenses on behalf of the patient in the past four weeks (>/= KD 51, or USD 173) had significantly higher scores than those who spent < KD 15(USD50.00), for the following subscales: (i) total objective burden (F = 3.4, df = 4/116, P < 0.01); (ii) tension (F = 7.3, P < 0.001); (iii) worrying (F = 3.0, P < 0.02); (iv) supervision (F = 3.9, P < 0.005); (v) subjective burden (F = 8.6, P < 0.001); and GHQ-12 (F = 5.9, P < 0.001).

### Relationship with patient's characteristics

#### Socio-demographic characteristics

Of the objective burden indices, age of patient was significantly negatively correlated with caregiver's urge subscale score (r = -0.193, P < 0.035). Patient's duration of illness was not significantly correlated with caregiver's GHQ-12 and IEQ subscale scores. The tendency for those caring for female patients to have higher burden and GHQ-12 scores, reached significance for the following: (i) worrying subscale (t = 2.9, df = 118, P < 0.004); and (ii) total objective burden score (t = 2.03, P < 0.045).

### Correlation with quality of care and psychopathology (Table 3)

**Table 3:** Significant correlations of caregiver burden indices and GHQ-12 scores with indices of patient's quality of care: N = 121

Variables	Pearson's r	P level
Total objective burden score (IEQ 16-35;37-42): Correlate with:		
Patient's basic met needs (CAN)	0.23	0.01
Patient's total met needs	0.21	0.02
Patient's basic unmet needs	-0.18	0.045

Subjective burden (IEQ 43): Correlate with		
Patient's basic unmet need	-0.199	0.029
Patient's health unmet need	-0.188	0.039
Patient's functioning unmet need	-0.229	0.01
Patient's total unmet need	-0.191	0.036

Total objective burden: Correlate with subjective QOL: LQLP:		
Patient's LQLP religion domain score	-0.37	0.001
Patient's LQLP health domain score	-0.25	0.006
Patient's LQLP perceived QOL score	-0.20	0.026
Patient's general well-being score	-0.31	0.001
Patient's negative affect	0.21	0.02

Subjective burden: Correlate with subjective QOL: LQLP:		
Patient's LQLP health domain score	-0.20	0.028
Patient's general well-being score	-0.29	0.001
Patient's negative affect	0.26	0.004

Total objective burden: Correlate with psychopathology scores:		
Patient's ICD-10 positive syndrome score	0.17	0.06
Patient's ICD-10 negative syndrome score	0.26	0.004
Caregiver GHQ-12 scores	0.31	0.001

Subjective burden: Correlate with caregiver GHQ-12 scores	0.36	0.001
Caregiver total objective burden score	0.49	0.001

Caregiver GHQ-12 score: Correlate with caregiver IEQ tension	0.39	0.001
Caregiver IEQ worrying	0.37	0.001
Caregiver IEQ supervision	0.28	0.002
Caregiver total objective burden score	0.31	0.001

In Pearson's correlation analyses, with indices of patient's quality of care and psychopathology as independent variables, the following patterns emerged: (i) while caregiver total objective burden correlated with patient's basic/total met needs for care (P < 0.01), subjective burden correlated negatively with patient's unmet need for care (P < 0.01). In other words, when caregivers sought to meet patients' needs, they experienced burden, but they were relatively free when patients apparently expressed no needs for care. In addition, caregiver burden was not significantly correlated with patient's perception of satisfaction with hospital services; (ii) caregiver burden was inversely correlated with various domains of patient's subjective QOL and general well - being, but directly correlated with patient's negative affect (P < 0.01); (iii) also, caregiver objective burden subscales and subjective burden were highly significantly correlated with their psychic distress (i.e., GHQ-12 score; P < 0.001); (iv) Of the patient's psychopathological indices, caregiver objective burden was only significantly correlated with the negative syndrome score (P < 0.004); (v) in the case of caregiver GHQ-12 score, the only significant correlation with indices of patient's quality of care was functioning met need for care (from CAN) (r = 0.21, P < 0.02).

### Tests of significance for association with indices of objective quality of life

Of the LQLP objective QOL indices assessed, only "going out for shopping" and "going for a ride" were significantly associated with burden scores. That is, caregivers felt relieved when patients attended these out door activities. Patient "going out for shopping" was significantly associated with lesser scores on caregiver's IEQ urge subscale (t = 2.4, df = 108, P < 0.018); worrying subscale (t = 3.1, P < 0.002); supervision subscale (t = 2.98, P < 0.004); and total objective IEQ score (t = 3.2, P < 0.002). The results for patient "going out for a ride" were similar.

### Multiple regression analyses (Table 4)

**Table 4:** Multivariate analyses of correlates of caregiver burden scores: stepwise regression analyses: final regression model

Dependent variables and significantly associated variables	%Variance(R^2^)	Standardized beta	T	P level	Tolerance*	VIF*
Dependent variable: total IEQ objective burden						
Education of caregiver	6.5	-0.17	1.7	0.09	0.91	1.1
Age of patient	6.6	-0.27	2.9	0.005	0.98	1.02
Patient's general well-being (subjective QOL: LQLP)**	7.7	-0.29	3.0	0.004	0.92	1.09
Patient "been out shopping" (objective QOL: LQLP)	14.2	0.38	4.0	0.001	0.98	1.00
	total = 34.9					

Dependent variable: global impression subjective burden						
Education of caregiver	9.3	0.25	2.48	0.15	0.93	1.07
Family income	5.0	0.18	1.87	0.066	0.97	1.03
Patient's negative affect	7.3	0.25	2.58	0.01	0.98	1.03
Patient's general well-being (LQLP)	4.9	-0.22	2.26	0.027	0.94	1.07
Patient "been out for a ride"	3.9:	0.19	2.07	0.04	0.99	1.01

	total = 30.4					
Dependent variable: IEQ supervision subscale score						
Patient's basic unmet need (CAN***)	5.0	-0.16	1.56	0.12	0.83	1.20
Patient's health domain QOL	5.5	-0.23	2.26	0.027	0.89	1.13
Patient's positive affect	4.9	0.17	1.60	0.114	0.80	1.25
Patient "been out shopping"	7.6	0.32	3.18	0.002	0.92	1.09

	total = 25.2					

* Tolerance (</= 0.2) and its reciprocal, VIF (>/= 4), are tests of multicollinearity between dependent and independent variables.

** LQLP: Lancashire Quality of Life Profile

*** CAN: Camberwell Assessment of Need

The independent variables entered were: (i) Step 1: caregiver and patient socio-demographic characteristics; (ii) Step 2: Patient's CAN and VSSS scores; (iii) Step 3: Patient's LQLP subjective scores, affect and self-esteem scores; (iv) Step 4: LQLP objective indices; (v) Step 5: patient's psychopathology (ICD-10 positive and negative syndrome) and GAF scores

In the final regression models, the following patterns emerged: (i) education of caregiver, patient's general well-being and patient participating in outdoor activities were significant predictors of total objective burden and subjective burden; (ii) of the IEQ subscale scores, education of caregiver was a significant predictor for tension and worrying. Age and sex of patient entered only the equations for urging and worrying, respectively. Taking patient out for shopping was a significant predictor of all subscales (5.5% - 13.3% of variance explained); while basic unmet need for care (5.0%) and health QOL domain score (5.5%) were only significant predictors of the supervision subscale. In other words, in the multivariate context, perceived higher burden was variously associated with caregiver lower level of education, patient's female gender and younger age, lower subjective QOL, expressed needs for hospital care, and not involving the patient in outdoor activities.

In an attempt to assess the objective burden contributors to the global rating of burden, we entered IEQ43 as dependent variable, and the following as independent variables: tension (minus IEQ43 score), worrying (minus IEQ43 score), supervision and urging subscale scores. The only significant predictor was the corrected tension subscale score (44.6% of variance explained, standardized beta = 0.67, t = 9.8, P < 0.0001). This indicates that the most problematic items of perceived burden belonged to the domain of tension, which consists of patient's difficult or disruptive behavior.

## Discussion

Inspired by the EPSILON report [29], and based on Schene's theoretical model [32], we used the responses of 121 family caregivers to explore a research question on the pattern of scores on IEQ-EU domains of caregiving experience and associated factors [12]. The highlights of our findings are that, the pattern of associations with domains of caregiving share similarities and differences with the international data that could be based on local cultural and mental health service factors. These findings are discussed from the perspectives of the international literature and their implications for the development of mental health care services in Kuwait.

### Pattern of domain scores

Judging by the IEQ mean subscale scores, majority of caregivers could be said to have experienced moderate levels of burden. The caregiving situation in Kuwait is similar to those of developing countries where the large family households (relative to those in developed countries) may have reduced burden levels because caring duties are shared in the large extended family system [22,44].

### Association of burden with caregiver characteristics

Of the caregiver characteristics investigated, the only significant relationships were that, higher caregiver burden was associated with low level of education, caregiver being either spouse or child of the patient, caregiver receiving welfare assistance and higher financial expenditure on behalf of the patient. The salience of caregiver education is one of the most replicated findings in this field [19-21]. Similar to our findings, an Australian study found that spouse caregivers and adult children of patients reported more burden than other caregivers [49]. The impact of financial expenditure shows the continuing salience of family out-of-pocket expenses for the care of patients in a country with generous national social welfare provisions for the citizens.

However, in contrast with reports indicating that higher burden was associated with more hours of contact with the patient [30,49], we found no significant relationship. This finding could be related to the social situation whereby the vast majority of Kuwaiti families have paid house-help living in the same household, thereby reducing the potentially negative impact of long hours of contact with the patient. Another view (not supported by our data) is that the large extended family system probably makes this item unreliable in our setting.

Despite the fact that majority (73.5%) admitted that the burden of caregiving was "fairly heavy"/"very heavy", over two-thirds felt that they had got used to the patient's problems and over three-quarters (81%) claimed hat they were able to cope with the problems. This is an interesting example of what has been described in the literature as "dissonance" in the experience of burden [6,50,51]. In a German study, it was noted that despite illness - related burdens, many spouses took positive stock of living together [3]. The all - pervading religious culture in Kuwait and the way the traditional extended family members are known to rally round sick members [52], would tend to support these positive caregiving attitudes.

### Relationship of burden with patient's characteristics

While the association of higher burden with patient's youth has much support in the literature [11,17,21,25,26], the finding that higher burden was experienced by those caring for female patients is uncommon [23]. However, the EPSILON study reported no significant difference by patient's gender [53]. In Kuwait, this could be explained by the well known fact that issues related to women are handled with secrecy in the Arab culture. Hence, it would be relatively more distressing if the female patient's behavior remained disruptive, especially as this would curtail her chances of marriage in a culture where traditionally arranged marriage is the order of the day. In non-Arab countries with a system of arranged marriage and with data on family burden (e.g., India) [27] the level of sexual segregation is much less strict than in Kuwait [52].

The correlation analyses indicated that higher caregiver burden was significantly associated with patient's met needs for care, diminished subjective QOL and negative symptoms of schizophrenia. Furthermore, caregiver psychic distress was associated with patient's formal met needs for care [17]. The interpretation of CAN results deserves a special attention because the study area has no community based interventions, and families have to take complete charge of patients' needs. We suggest that the implication is that, for this sample, the number of met needs would be a direct indicator of family involvement, whereas the number of unmet needs could possibly reflect family disengagement. Thus, the interpretation of the results should be completely different from that of previous European reports (where services contributed to the meeting of patient's needs). Although needs cause burden because families have to ensure that these needs are met, the presence of many met needs could indicate more psychopathology and may also imply that the patient has social support [17]. While the relationship of burden with service satisfaction is controversial [15], the lack of a significant relationship in our study could be attributed to the uniformity of experience in a centralized service that is totally free - of - charge and lacks community-based services.

Our findings about the association of burden with psychopathology and diminished subjective QOL have much support in the literature [18,26,54]. However, there is no information on the relationship of objective QOL with caregiver burden. In this regard, our finding that taking the patients out for outdoor activities was associated with reduced caregiver burden is noteworthy. This underscores the need for provision of community - based programs that will emphasize outdoor activities in our service. Finally, the results of the regression analysis with subjective burden as dependent variable and objective burden subscale scores as independent variables, gave support to the widely noted observation that the most important determinant of perceived burden is the patient's disruptive or difficult behavior [22,23,55].

### Limitations and strengths of the study

The major limitations of the study are that it was cross-sectional, and the participants were not representative of the general population of schizophrenia caregivers in Kuwait, especially as only one family member was interviewed [13]. However, we have used an internationally validated questionnaire that was found to have adequate reliability and validity indices in our setting (data to be presented elsewhere). This has made our findings comparable with the international reports. In addition, we have added the following new perspectives to the analysis of the IEQ: (i) defining IEQ43 as subjective burden [3] and using it as a dependent variable; and (ii) analyzing the relationship of indices of objective QOL with burden. We note that IEQ46 was not framed in such a way as to determine the direction of the presumed change in emotional relationship (i.e., positive or negative change). It would be useful to make that clarification because of the known impact of emotional relationship with the patient on caregiver's health [56].

## Conclusion

Our results indicate that, despite differences in service set - up and culture, the IEQ-EU can be used in Kuwait as it has been used in the western world, to describe the pattern of scores on the dimensions of caregiving. Hence, we have widened the cross-cultural base of the evidence that the IEQ dimensional structure of caregiving reflects universal experience of caregiving [30]. We suggest that, differences with the international data reflect peculiarities of culture and type of service. Despite generous national social welfare provisions, experience of burden was the norm and was significantly associated with patient's disruptive behavior. The results underscore the need for provision of community - based programs in our setting, and continued intervention with the families in order to improve the quality of care [5,54].

## Competing interests

The authors declare that they have no competing interests.

## Authors' contributions

MAZ and JUO designed the study, analyzed the data and prepared the manuscript. MAZ supervised data collection. All the authors read the manuscript and approved it.

## Pre-publication history

The pre-publication history for this paper can be accessed here:

http://www.biomedcentral.com/1471-244X/10/71/prepub
